# Supplementary material for: A guide to selecting high-performing antibodies for Optineurin (UniProt ID: Q96CV9) for use in western blot, immunoprecipitation, and immunofluorescence
Source: F1000Res. 2025 Oct 17;14:1137. [Version 1] doi: 10.12688/f1000research.169966.1 (PMC12914170; doi:10.12688/f1000research.169966.1)
Supplement: Peer review history

# Reviewer report: Reviewer response for version 1

**DOI:** 10.5256/f1000research.187366.r425570

**Published:** 2026-02-17

**Authors:** Andrew Buchanan

**Affiliations:** 1 Stealth Biotech, Cambridge, Cambridgeshire, UK

This is a clear and well set put example of antibody quality control for a range of experiments. The data is straight forward to interpret from the figures and tables. This enables the reader to have a well informed choice of what antibody to take into their experimental setting for further validation.

 A minor point is that for DepMap the authors say > 2.5 log2 is a suitable cut-off, but they choose U2OS with 0.89 log2 TPM+1. However there was enough protein expression for the validation work.

Are sufficient details of methods and materials provided to allow replication by others?

Yes

Is the rationale for creating the dataset(s) clearly described?

Yes

Are the datasets clearly presented in a useable and accessible format?

Yes

Are the protocols appropriate and is the work technically sound?

Yes

Reviewer Expertise:

Therapeutic antibody discovery and early development

I confirm that I have read this submission and believe that I have an appropriate level of expertise to confirm that it is of an acceptable scientific standard.

# Reviewer report: Reviewer response for version 1

**DOI:** 10.5256/f1000research.187366.r440395

**Published:** 2025-12-30

**Authors:** Deborah Moshinsky

**Affiliations:** 1 Institute for Protein Innovation, Boston, Massachusetts, USA

The authors tested 8 commercially available antibodies against optineurin (OPTN) for activity in Western Blot, Immunoprecipitation, and Immunofluorescence as part of a larger ongoing effort to address antibody reproducibility issues in Science.  They first made knockout cells using a cell line that was shown to express the target.  Then they ran bespoke assays using each of the 8 antibodies.  Results were shown clearly.  The authors describe briefly why they do not engage in result interpretation but did provide general guidance on interpreting data in their assays.  Readers can surmise from the data which antibodies should work well in their applications of choice.

 For the choice of cell line, it is stated that cell lines that express the target at levels greater than 2.5 log _2_ (transcripts per million “TPM” + 1) have been found to be a suitable cut-off.  However, U2OS expresses the protein optineuron transcript at less than 2.5 (0.89 log _2_ TPM+1).  Please explain or note that this is an outlier.  Also, as minor points 2 typos were identified in the article.  "performanyce" is present in the paragraph starting with 'In conclusion' after Figure 3.  Also, in the abstract, the word 'tested' is missing from the sentence, "Here we have eight optineurin commercial antibodies for western blot..."

Are sufficient details of methods and materials provided to allow replication by others?

Yes

Is the rationale for creating the dataset(s) clearly described?

Yes

Are the datasets clearly presented in a useable and accessible format?

Yes

Are the protocols appropriate and is the work technically sound?

Yes

Reviewer Expertise:

Antibody characterization and validation

I confirm that I have read this submission and believe that I have an appropriate level of expertise to confirm that it is of an acceptable scientific standard.
